# De Novo Isolation & Affinity Maturation of yeast-displayed Virion-binding human fibronectin domains by flow cytometric screening against Virions

**DOI:** 10.1186/s13036-019-0203-2

**Published:** 2019-10-16

**Authors:** Pete Heinzelman, Alyssa Low, Rudo Simeon, Gus A. Wright, Zhilei Chen

**Affiliations:** 1 Department of Microbial Pathogenesis & Immunology, Texas A&M University, College Station, Texas 77843 USA; 2 Department of Veterinary Pathobiology, Texas A&M University, College Station, Texas 77843 USA

**Keywords:** Flow cytometry, Zika virus, Human Immunodeficiency Virus, AIDS, yeast display, fibronectin, protein engineering, antibody engineering, directed evolution, phage display

## Abstract

**Background:**

The promise of biopharmaceuticals comprising one or more binding domains motivates the development of novel methods for de novo isolation and affinity maturation of virion-binding domains. Identifying avenues for overcoming the challenges associated with using virions as screening reagents is paramount given the difficulties associated with obtaining high-purity virus-associated proteins that retain the conformation exhibited on the virion surface.

**Results:**

Fluorescence activated cell sorting (FACS) of 1.5 × 10^7^ clones taken from a naïve yeast surface-displayed human fibronectin domain (Fn3) against whole virions yielded two unique binders to Zika virions. Construction and FACS of site-directed binding loop mutant libraries based on one of these binders yielded multiple progeny clones with enhanced Zika-binding affinities. These affinity-matured clones bound Zika virions with low double- or single-digit nanomolar affinity in ELISA assays, and expressed well as soluble proteins in *E. coli* shake flask culture, with post-purification yields exceeding 10 mg/L.

**Conclusions:**

FACS of a yeast-displayed binding domain library is an efficient method for de novo isolation of virion-binding domains. Affinities of isolated virion-binding clones are readily enhanced via FACS screening of mutant progeny libraries. Given that most binding domains are compatible with yeast display, the approach taken in this work may be broadly utilized for generating virion-binding domains against many different viruses for use in passive immunotherapy and the prevention of viral infection.

**Electronic supplementary material:**

The online version of this article (10.1186/s13036-019-0203-2) contains supplementary material, which is available to authorized users.

## Background

Recombinant protein therapeutics comprised of multiple distinct virion-binding domains, e.g., human antibody fragment variable regions (Fvs) or camelid antibodies (VHHs), have shown remarkable efficacy as broadly neutralizing agents of viral infectivity in animal models of human influenza [1] and HIV [2]. Expansion of the existing pool of virion-binding domains and the development of methods to produce the virion-binding domains at high expression levels in the comparatively simple host system of *E. coli* would aid efforts to make better, more affordable multispecific antivirals.

The discovery of binders/neutralizers of virions is often challenging due in large part to the membrane-bound nature of viral envelope proteins, making them difficult to express recombinantly in a purified form at high levels. It is possible that extracellular segments of viral envelope proteins can be expressed as soluble proteins/peptides for binder discovery, but these “out-of-context” proteins/peptides might not faithfully recapitulate the secondary/tertiary structure present on the viral surface. These considerations make isolation and engineering of virion-binding domains by screening against intact virions a more desirable approach to obtaining pools of virion-binding domains than multiplex screening against collections of recombinantly expressed virion surface proteins/peptides. The most recent body of reported work involving de novo isolation of surface-displayed binder libraries appears to use phage-displayed libraries screening against immobilized virions amenable to high titer in vitro production [3–5].

This paucity of precedent for successful screening of virus-targeting protein libraries is likely attributable to the substantial numbers of highly purified virions needed to adequately coat the surfaces of immunotubes or magnetic particles typically used in large scale screening of surface-displayed protein libraries. Virion preparations with high purity and high concentration must be used for immunotube or magnetic particle coating to minimize deposition of contaminating proteins on the surfaces of these solid screening supports; such deposition can lead to the isolation of Abs and Ab analogues that bind to epitopes presented by these protein contaminants rather than the target virions. In the above noted examples, the two naïve mouse scFv library screening articles used immunotubes coated with fifty micrograms of virions, an astonishing amount of virions, for the first round of phage display [3, 4]. It is reasonable to posit that coating of magnetic particles needed for a first round screen of this same naïve scFv library would require a similar mass of virions.

Generating fifty micrograms of highly purified, clinically relevant virions such as HIV, influenza, or Zika using the cell culture-based methods typically employed [6, 7] for producing virions to be used in research settings could in some cases require harvesting virions from tens of liters of cell culture volume. An additional challenge associated with obtaining large quantities of highly purified virions is the need to use affinity chromatography [8–10] for virion purification. Although affinity chromatography methods can provide high purity virions with yields exceeding 90 %, these approaches require upstream concentration of virus-containing cell culture media. Upstream concentration is laborious in and of itself and has been thoroughly developed and optimized for only a small number of viruses.

**Fig. 1:**
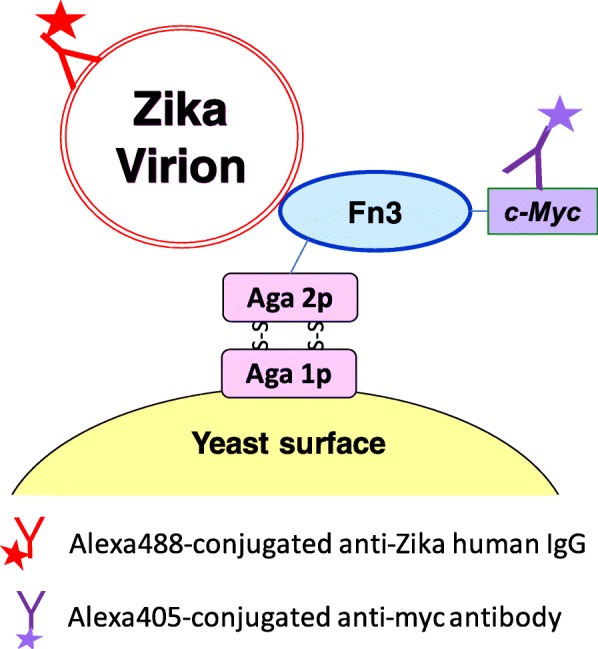
Schematic for sandwich detection of yeast surface-displayed Fn3 binding to Zika virions. Anti-Zika human IgG conjugated with Alexa488 enables detection of Fn3-Zika virion binding interaction. Alexa 405-conjugated anti-*myc* antibody (IgY from chicken) binds to *myc* tag on Fn3 C-terminus and allows quantification of Fn3 surface display

Fluorescence activated cell sorting (FACS)-based approaches, particularly those that incorporate the yeast surface display screening platform [11], typically require less target for binder discovery than solid-phase panning methods. As such, virions that have been obtained by broadly applicable lab-scale purification methods such as centrifugal ultrafiltration (UF) and density gradient ultracentrifugation (UC) can be used for FACS-based discovery of virion binders. The need for less virus for virus-binder discovery using FACS arises from the sensitive method of detection used, as depicted in Fig. 1. The use of fluorescently-conjugated commercially available immunoglobulin Gs (IgGs) that are specific for the target virions results in a fluorescence signal for yeast cells that are displaying virion-binding scFvs or Ab analogues whereas yeast that display scFvs or Ab analogues that bind to contaminants in the virion preparations are non-fluorescent. Importantly, FACS allows the interrogation of both positive and negative binding events on a single-cell basis.

Although the majority of published methods for de novo isolation of soluble protein target antigen binding scFvs and Fn3s [12–14] from naïve yeast surface-displayed libraries are based on using magnetic particles to oversample libraries with clonal diversities approaching or exceeding one-billion, there is underappreciated precedent for de novo isolation of soluble protein target-binding scFvs from pools of approximately 10^7^ naïve scFv clones via FACS. Furthermore, recent development of immune libraries with paired heavy and light chain repertoire enabled the selection of high affinity binders from a very small library (~ 10^5^) [15]. These considerations, combined with the desire to establish a de novo virion-binding scFv or Ab analogue isolation method that requires only relatively modest numbers of virions, i.e., 10^10^ - 10^12^, enriched via convenient methods such as UF or UC, motivated our pursuit to select virus binders from approximately fifteen-million yeast cells.

In this study, a yeast-displayed naïve Fn3 library [13] was used as the model library to demonstrate the feasibility of enriching binders to whole virions. In part by virtue of their being devoid of disulfide bonds, Fn3s can be easily expressed in *E. coli*, making it simple to obtain large quantities of soluble, purified Fn3 protein needed for assessment of virion-binding Fn3 properties in binding studies, e.g., ELISA assays, and virus infectivity neutralization assays. However, it should be noted that our method can be extended to any binder libraries (e.g. single chain fragment variable regions (scFv) [11], nanobodies [16], designed ankyrin repeat proteins (DARPins) [17], etc) compatible with yeast display.

Our choice of Zika virus as the target for this study was motivated by its clinical relevance. Infection of humans by the Zika virus, which is a flavivirus transmitted by mosquitos, can lead to brain damage and in some cases can even be fatal [18]. Given that there is no effective Zika virus vaccine and local outbreaks occurred in the states of Florida and Texas during 2016 and 2017 there is clearly a need to develop novel agents for preventing and treating Zika infections.

## Results

### De novo isolation of Zika Virion-binding Fn3s

Isolation of Zika virion-binding Fn3s from the yeast-displayed Fn3 library noted above [13] began with flash sorting of a fifteen-million clone sample of the library (Fig. 1). Fn3-displaying yeast library was incubated simultaneously with concentrated Zika virions in PBS the presence of Alexa488-conjugated anti-Zika human IgG as well as Alexa405-conjugated anti-Myc antibody. Yeast population positive for both Alexa488 and Alexa405 signals was harvested. In the second and third rounds of FACS, a simultaneous forward/counterscreening approach was employed to prevent enrichment of human IgG-binding Fn3 library clones. Briefly, in addition to the above-mentioned antibodies, a third isotype control human IgG, conjugated with Alexa647, was included in the mixture. Yeast populations negative of Alexa647 signal and positive for both Alexa488 and Alexa405 were harvested (Additional file 1: Figure S1).

**Fig. 2:**
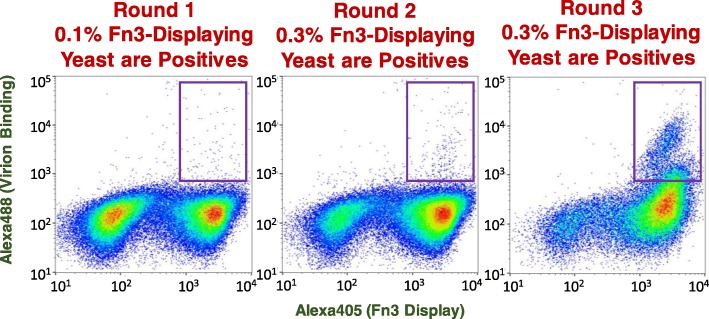
Flow cytometry dot plots for FACS enrichment of Zika virion-binding Fn3s. Y-axes denote binding of surface-displayed Fn3s to Zika virions as quantified via sandwich detection using Alexa488-conjugated anti-Zika IgG. Panels, left-to-right, show representative samples (100,000 yeast cells) of yeast populations screened during FACS rounds one, two and three

As illustrated in Fig. 2, three rounds of FACS was adequate to enrich Zika virion-binding Fn3s more than 100-fold relative to the starting naïve Fn3 library. Flow cytometric analysis of the yeast population screened in round three of FACS (Fig. 2, right panel) after these yeast were incubated with Alexa488-conjugated anti-Zika IgG sans any preceding incubation with Zika virions revealed a dramatic decrease in the number of Alexa488-positive yeast; fewer than 2 % of the Fn3-displaying yeast were Alexa488 positive in this analysis (data not shown). This result strongly suggested that the forward/counterscreening strategy utilized in FACS rounds two and three was effective in preventing the enrichment of Fn3s that bind to anti-Zika IgG rather than Zika virions.

**Fig. 3:**
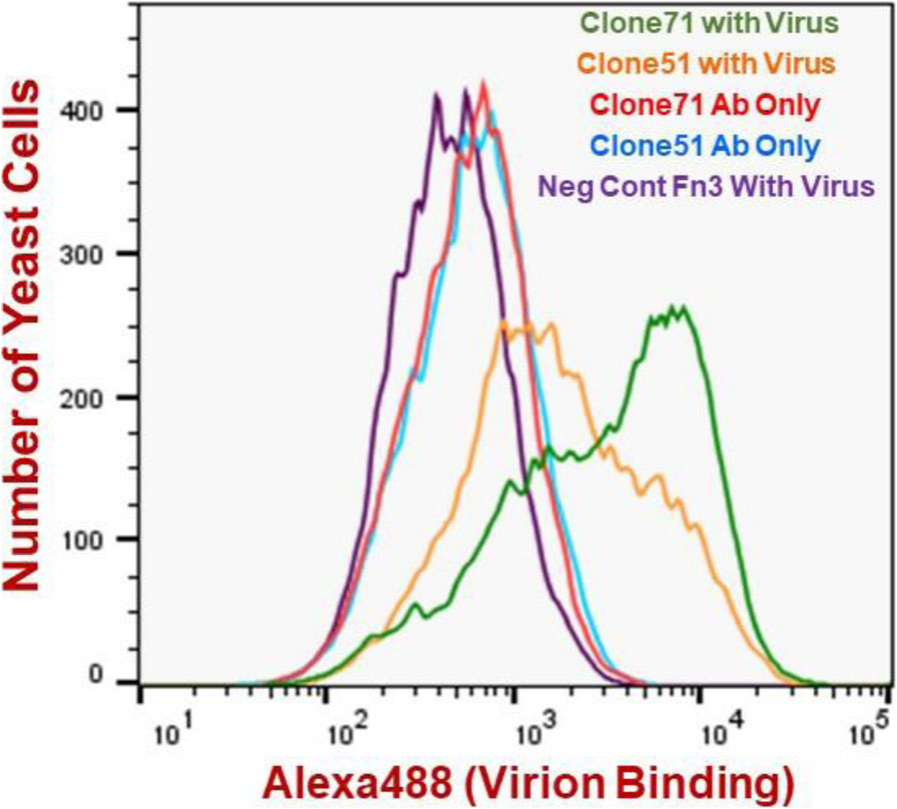
Flow cytometric histogram overlay for yeast-displayed Fn3s binding to Zika virions. X-axis denotes binding of surface-displayed Fn3s to Zika virions as quantified via sandwich detection using Alexa488-conjugated anti-Zika IgG. Ab only denotes yeast samples not incubated with Zika virions prior to labeling with Alexa488-conjugated anti-Zika IgG secondary reagent. Histograms contain data for 30,000 analyzed yeast cells per sample

Sequencing of twenty individual Fn3 clones, which were isolated by plating a fraction of the yeast population enriched in round three of FACS on solid media, revealed two unique Fn3 sequences; both of these sequences were full-length Fn3s devoid of any insertions, frameshifts or deletions (Additional file 1: Text S1). As depicted in Fig. 3, both yeast populations displaying these Fn3s were Alexa488 positive after serial incubation with ultrafiltered, buffer-exchanged Zika virion-containing supernatant from Vero cells and Alexa488-conjugated anti-Zika IgG (with Virus). Yeasts displaying these two Fn3s were Alexa488 negative following serial incubation with respective ultrafiltered, buffer-exchanged culture supernatant obtained from uninfected Vero cell cultures and anti-Zika IgG (Ab Only). This outcome indicates that the Fn3s bound to Zika virions rather than contaminants in the Zika virion preparations or anti-Zika IgG.

Zika virion binding signal for one of the two Fn3s, Clone71, was distinctly higher than that for its virion-binding counterpart, Clone51 (Fig. 3). Given this apparently increased virion binding affinity Clone71 was chosen as the parental Fn3 for subsequent affinity maturation.

### Affinity maturation of Clone71 Zika Virion-binding Fn3

**Fig. 4:**
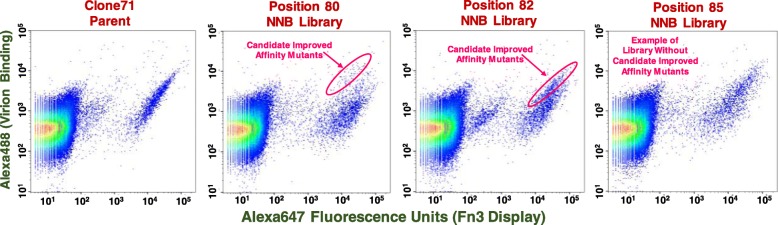
Representative flow cytometric dot plots for analysis of yeast-displayed Clone71 site-directed saturation mutant libraries. Dot plot data was used to identify libraries containing candidate improved affinity Fn3 mutants. Pink ellipses in dot plots for position 80 and position 82 saturation mutant libraries encase yeast populations lying above the bulk diagonal. Position 85 mutant library (rightmost panel) provides example of saturation mutant library devoid of candidate higher affinity virion binders. Induced yeast were mixed with uninduced yeast at a ratio of 1:10 to prevent ligand (Zika virion) depletion effects. Dot plots contain data for 250,000 analyzed yeast cells

Engineering of Clone71 progeny mutants with increased Zika virion binding affinity began with construction of twenty-three single site-directed saturation mutagenesis libraries in which the native codon at each of the positions comprising the three Clone71 Fn3 binding loops (Additional file 1: Text S1) was replaced with a degenerate NNB codon. The representative dot plots of Fig. 4 show that introducing mutations at two of the twenty-three binding loop positions, Phe80 and Ile82, gave rise to populations of Clone71 mutants with Zika virion binding fluorescence signals that were notably greater than signals for both the wild type Clone71 parent Fn3 and the majority of the Clone71 mutants contained within these respective saturation mutant libraries.

Prior to enriching high-affinity virion-binding clones from the position 80 and position 82 mutant libraries via FACS a double site-directed Clone71 saturation mutant library containing NNB codons at positions 80 and 82 was constructed for the purpose of seeking to capture additive or synergistic increases in binding affinity that may arise from simultaneous mutagenesis of these two binding loop positions. The FACS dot plots of Figure S3 (Additional file 1) illustrate that all three saturation mutant libraries contained Clone71 progeny mutants with Zika virion binding affinity greater than that observed for the Clone71 parent. The approximate percentages of increased affinity Clone71 progeny mutants ranged from one to three with the position 80 single site saturation mutant library containing the greatest percentage of higher affinity clones.

Plating FACS-enriched yeast populations on solid media and sequencing of seven individual Fn3 mutant clones from each of the three enriched clonal pools revealed F80P and I82Y as dominant mutations. F80P was present in seven of seven position 80 library clones, I82Y was present in four of seven position 82 library clones, and the F80P/I82Y combination was carried by three of seven position 80/position 82 double site-directed mutant library clones. None of the other position 82 or double mutant library Fn3 clones identified by sequencing were represented more than once.

**Fig. 5:**
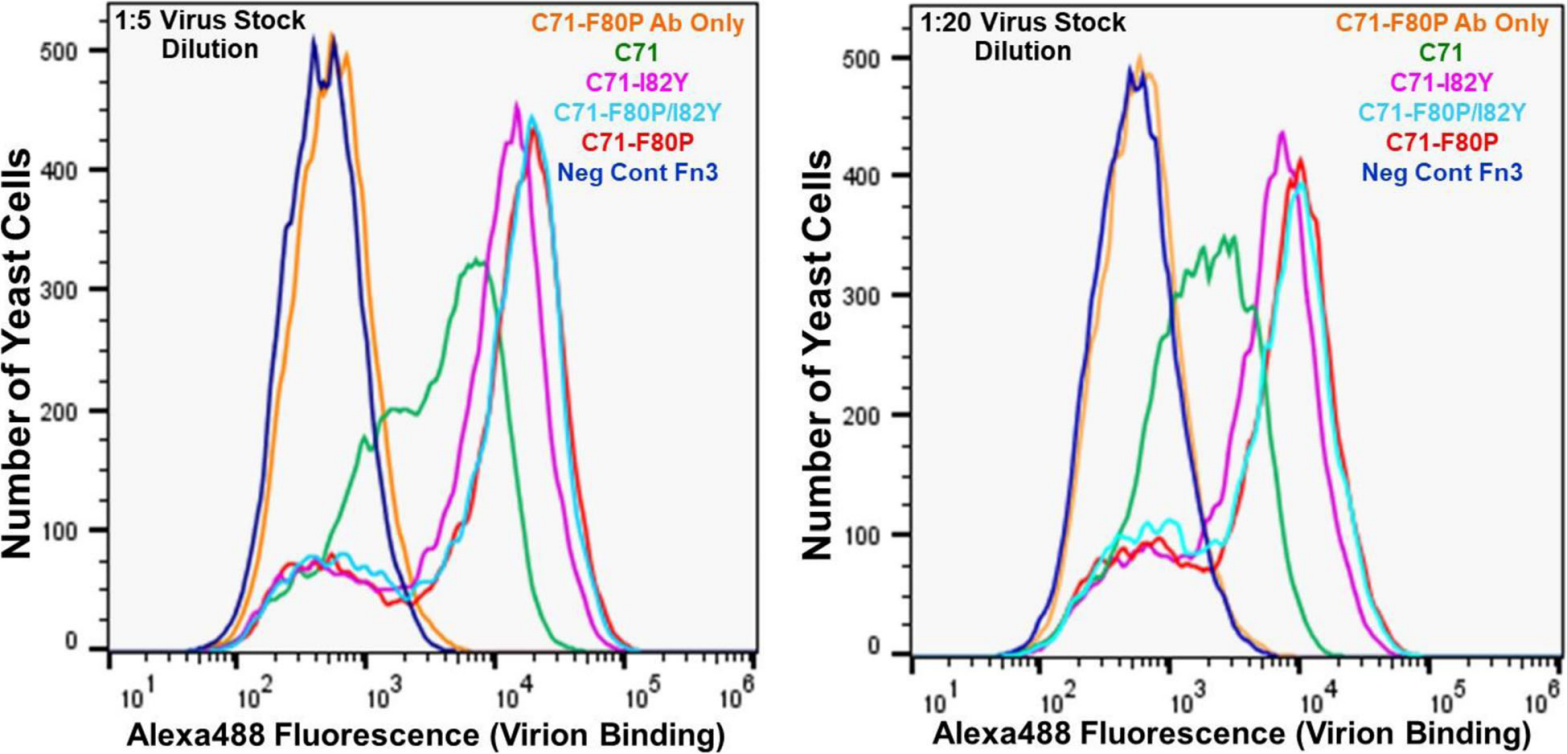
Flow cytometric histogram overlays for yeast-displayed Fn3s binding to Zika virions. X-axes denote binding of surface-displayed Fn3s to Zika virions as quantified via sandwich detection using Alexa488-conjugated anti-Zika IgG. C71: Clone 71. Ab Only denotes yeast samples incubated with culture supernatant from naïve Vero cells prior to labeling with Alexa488-conjugated anti-Zika IgG secondary reagent. Left panel: Yeast incubated with Zika virion stock diluted 1:5 in PBS/0.3% BSA. Right panel: Yeast incubated with Zika virion stock diluted 1:20 in PBS/0.3% BSA. Histograms contain data for 30,000 analyzed yeast cells per sample

Flow cytometric analysis of individual yeast-displayed Clone71 mutant progeny clones binding to Zika virions, as depicted in the histograms of Fig. 5, showed that the F80P, I82Y and F80P/I82Y mutants had similar Zika virion binding affinities; the I82Y mutant binding signal was slightly less than that for the F80P and F80P/I82Y mutants. All three mutants possessed notably greater virion binding affinity than the Clone71 parent Fn3 and flow cytometric analysis carried out using negative control concentrated culture supernatant from uninfected Vero cell cultures (Ab only), prepared by ultrafiltration (UF) as described in the Materials & Methods section, strongly suggests that these increases in affinity are due to increased binding to Zika virions rather than binding to other constituents present in the UF Zika virion preparations.

### Soluble production of Zika Virion-binding Fn3s

Zika virion-binding Fn3 affinity maturation was followed by Fn3 expression in *E. coli* shake flask cultures and subsequent immobilized metal affinity chromatography (His_6_-tag/Ni-NTA) purification of the solubly expressed proteins. Post-purification Fn3 yields ranged from 10 to 50 mg/L of shake flask culture with the Clone71 F80P and F80P/I82Y mutants having lower yields than the other Fn3s.

**Fig. 6:**
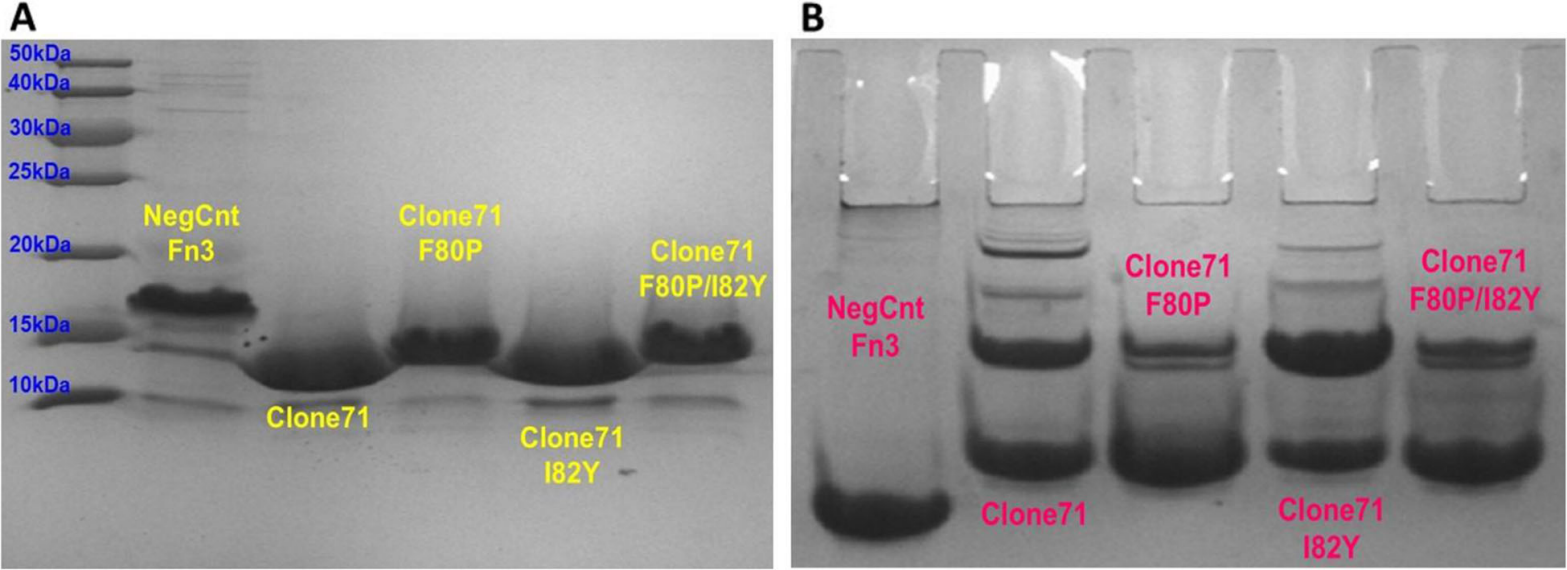
Denaturing (**a**) and native (**b**) PAGE analyses of Zika-binding and negative-control Fn3s. Calculated molecular weight for Fn3s is ~ 13 kDa. Native PAGE analysis indicates that the Clone71 F80P mutant reduces the formation of dimeric and oligomeric Fn3 complexes

Clone71, the three progeny mutants, and a negative control Fn3 that does not bind to Zika virions all ran near the anticipated molecular weight of ~ 13 kDa in the denaturing (SDS) PAGE analysis appearing in Fig. 6a with F80P mutant run at a higher apparent molecular weight. Since Fn3s do not contain disulfide bonds, the small differences in apparent molecular weight are likely due to differing susceptibilities to denaturation by SDS. The observed presence of monomeric, dimeric, and oligomeric Clone71 and Clone71 mutant progeny complexes in the native PAGE analysis of Fig. 6b is unsurprising given the numerous reports of purified soluble Fn3s existing in both monomeric and higher order states [19, 20]. The F80P mutation clearly increases the relative fraction of monomeric Fn3 appearing in the native PAGE analysis when compared to the parental Clone 71.

### Binding of purified, soluble Fn3s to Zika Virions in ELISA assays

**Fig. 7:**
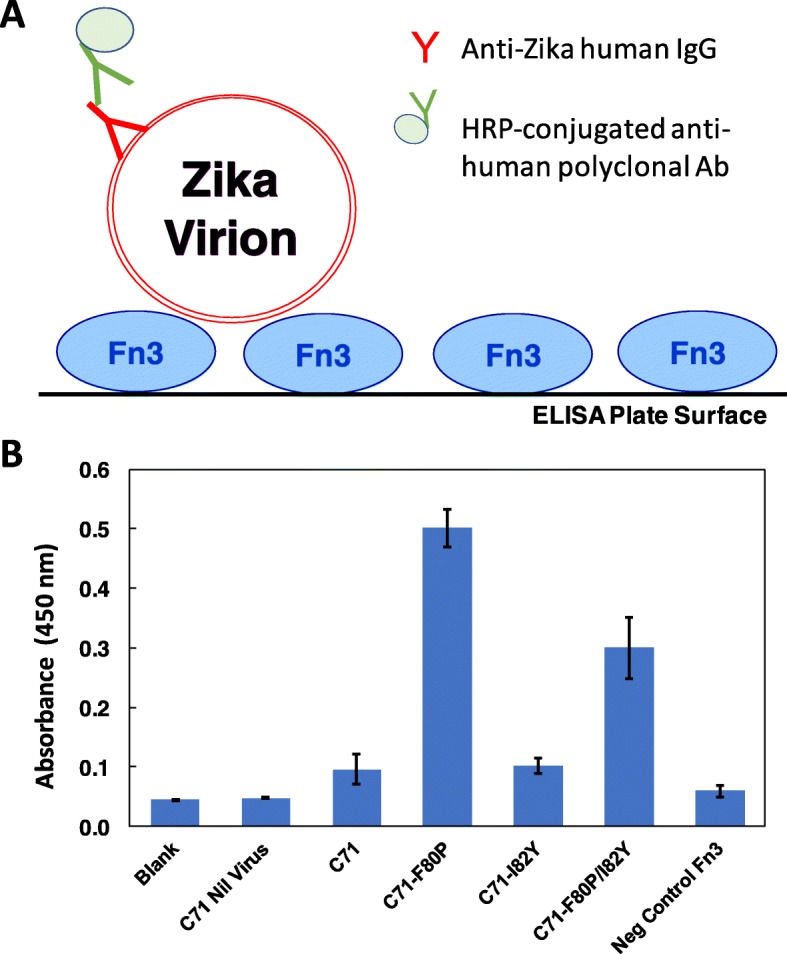
Immobilized Fn3 ELISA assay. **a** Assay schematic. **b** Assay absorbance values. C71: Clone 71. ‘C71 Nil Virus’ denotes negative control in which ELISA plate-immobilized C71 Fn3 was incubated with UF conditioned culture media supernatant from naive Vero cells. Error bars denote standard deviations for triplicate absorbance measurements

Initial assessment of purified Fn3s binding to Zika virions in UF virion preparations was performed using an ELISA assay in which purified Fn3s were immobilized on the ELISA plate and sandwich detection with anti-Zika human IgG was employed to quantify interactions between immobilized Fn3s and Zika virions. This assay format, which is shown in Fig. 7a, rather than an assay format in which free in solution Fn3s are allowed to bind to immobilized Zika virions, was used because given the large size, i.e., diameter of approximately 50 nm, of Zika virions [21] relative to Fn3s, which have a hydrodynamic radius of less than 5 nm [22], there is a potential opportunity for avidity effects, which could arise from single Zika virions simultaneously being bound by more than one immobilized Fn3, to be leveraged in achieving high assay sensitivity.

The results of Fig. 7b illustrate that immobilized Clone71 binds to Zika virions but does not yield a binding signal when incubated with virion-null negative control UF Vero cell culture supernatant. Both Clone71-F80P and F80P/I82Y mutants showed greater virion binding affinity than the parental Clone71. Surprisingly, the I82Y mutation, which markedly increased binding affinity in the yeast-displayed Fn3 flow cytometry virion binding analyses, did not have a significant effect on virion binding affinity in this immobilized Fn3 ELISA assay.

**Fig. 8:**
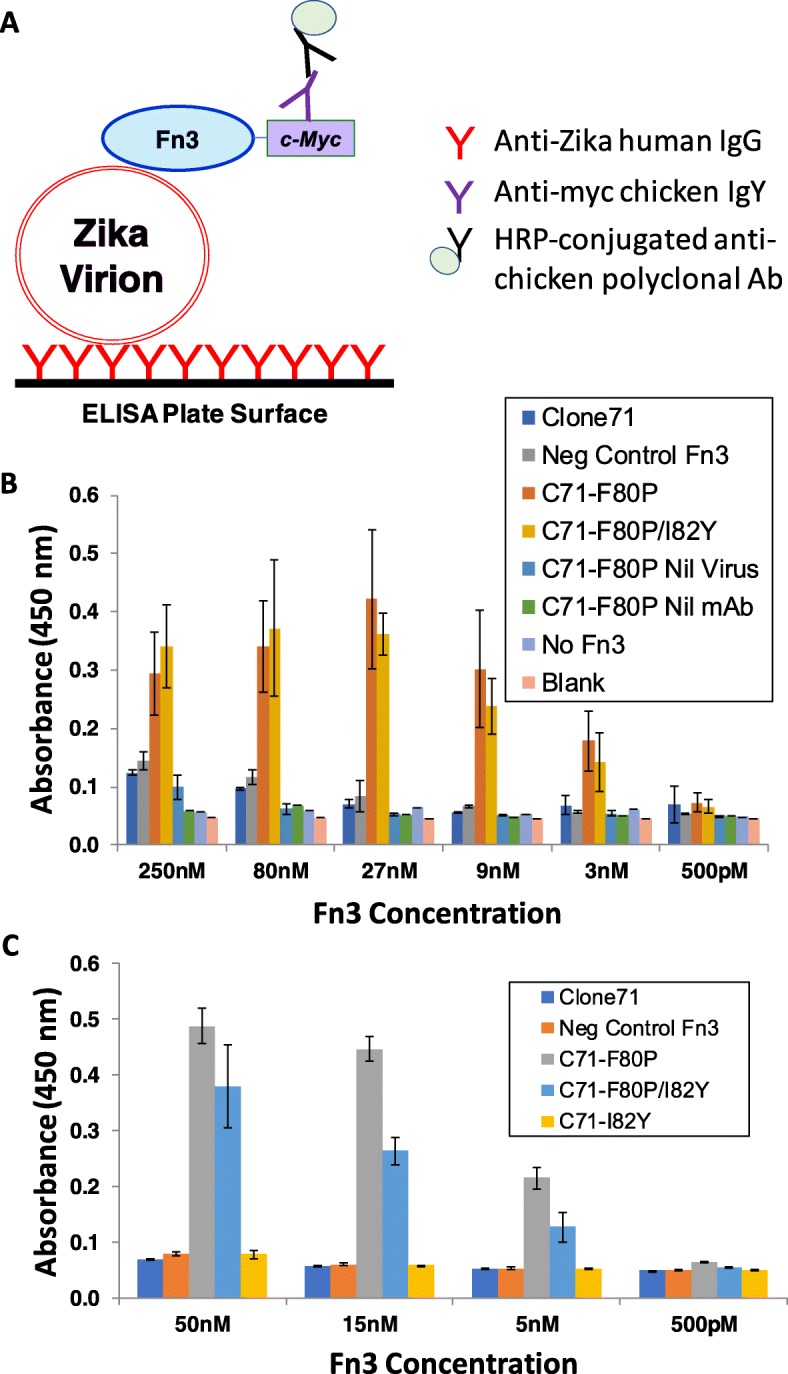
Free-in-solution Fn3 ELISA assay. **a** Assay schematic. **b**, **c** Sandwich detection of virions bound by immobilized IgG. ‘Nil Virus’ denotes incubation with blank assay buffer, rather than virions, prior to incubation with Fn3. ‘Nil mAb’ denotes coating of ELISA plate assay wells with BSA rather than anti-Zika IgG. Error bars denote standard deviations for duplicate absorbance measurements

A second ELISA assay format that measures binding of free in solution Fn3s to immobilized Zika virions (Fig. 8) was also tested. Although this free-in-solution Fn3 assay configuration does not offer the potential to leverage avidity effects in enhancing binding interaction detection sensitivity, as is the case for immobilized Fn3 ELISA assays, it enables one to accurately titrate the amount of soluble Fn3 across wells of the assay plate and thus quantitatively estimate the strength of Fn3-Zika virion binding interactions.

Contaminating molecular species, particularly proteins, present in virion preparations can occupy immobilization sites on the ELISA plate surface and thus reduce the surface density of immobilized virions. In light of this observation and the challenge, the ELISA plate was first coated with the high purity anti-Zika IgG and then incubated overnight at 4 °C with UF Zika virion preparations. The data of Fig. 8b mirror the results of the immobilized Fn3 ELISA assay (Fig. 7) in showing that the F80P mutation markedly increases Clone71’s binding affinity toward Zika virions. Importantly, respective negative control wells coated with either bovine serum albumin (Nil mAb) rather than anti-Zika IgG or incubated with virion-null UF culture supernatant (Nil Virus) do not show any Fn3 binding signal; this result is in agreement with the absence of Fn3 binding signal observed for the virion-null control wells in the immobilized Fn3 ELISA assay of Fig. 7.

In contrast to the immobilized Fn3 ELISA assay, where virion binding signal was observed for the wild type Clone71 and the I82Y progeny mutant, neither of these Fn3s produced binding signals above that for the negative control Fn3 in the free-in-solution ELISA assay. This result is in accord with the observed lower binding signals for these two Fn3s relative to F80P mutation-containing Fn3s in the immobilized Fn3 ELISA assay and supports the existence of the above posited increase in assay sensitivity for the immobilized Fn3 ELISA configuration relative to the free in solution Fn3 ELISA format.

Taken together, the ELISA results presented in Fig. 8b and c, where the latter ELISA featured Zika-binding Fn3s titrated across a narrower and lower range of Fn3 concentrations to better enable accurate estimation of binding affinities, indicate that the Clone71 mutants F80P and F80P/I82Y bind to Zika virions when present in solution at concentrations in the low nanomolar range.

## Discussion

In this report we demonstrate that flow cytometric sorting of small samples of highly diverse yeast-displayed Fn3 libraries is an effective method for de novo isolation and affinity maturation of virion-binding protein domains. Specifically, flow cytometric sorting was performed on only 1.5 × 10^7^ clones derived from a Fn3 library with a clonal diversity exceeding 4 × 10^9^, yielding two unique Zika virion binders that were subsequently subjected to affinity maturation to obtain variants with single-digit nanomolar virion binding affinity.

It is conceivable that FACS enrichment of yeast-displayed virion-binding domains possessing affinity toward multiple virus strains would be facilitated by screening against respective virion strains that have been covalently appended with different fluorescent dyes via standard N-hydroxysuccinimide ester (NHS) coupling chemistry [22, 23] and/or using fluorescently-labeled IgGs that are specific for particular virus strains as secondary detection reagents. Given that widely available FACS apparatuses can simultaneously detect photon emissions from double-digit numbers of different fluorophores [24, 25] one would not anticipate there being any substantial obstacles to the above described multicolor labeling approach being implemented by researchers who wish to perform yeast surface display flow cytometry experiments that feature virions as target antigens.

Clone 71, as well as all the progeny mutants, exist in both monomeric and multimeric state (Fig. 6b). Fn3 dimerization and/or oligomerization have been observed to reduce or abolish the ability of Fn3’s to bind their target antigens [19, 20]. Noteworthy here is that dimerization and/or oligomerization and the corresponding potential for reductions in or loss of target binding affinity do not negate the value of pursuing Fn3s as virion-binding domains for incorporation into multispecific antiviral biopharmaceuticals. Supporting this statement are the numerous reports of rational design approaches [20, 26, 27] which have successfully identified mutations that increase the thermostability of both Fn3s and structurally similar target antigen-binding domains derived from the human Tenascin-C protein. Such thermostability increases have been observed to reduce the occurrence of intramolecular Fn3 domain rearrangements that frequently precede the association of Fn3 monomers into dimeric or oligomeric states [20, 26]. In the case of Fn3 variants F80P, I82Y and F80P/I82Y, their melting temperature was determined using the ThermoFlour method [28, 29]. All these Fn3 variants appear to be highly thermostable with T_m_ > 95 °C. This result is not particularly surprising as there are a number of reports of engineered Fn3s with very high thermostability [26, 30].

The free-in-solution Fn3 ELISA assay results of Fig. 8 speak strongly to the utility of FACS for increasing the binding affinities of virion-binding Fn3 clones after such clones have been isolated from naïve yeast surface-displayed libraries. Particularly striking is the ability of the Clone71 F80P mutant to bind Zika virions when present in solution at single-digit nanomolar concentrations (Fig. 8c) whereas the Clone71 parent did not exhibit detectable virion binding at concentrations as high as 250 nM.

Unfortunately, none of the isolated Fn3s exhibit Zika virus-neutralization activity (data not shown). This result is not particularly surprising given that less than 5 % of the IgGs isolated from flavivirus-infected or flavivirus-vaccinated humans possess virus-neutralization activity [21]. Further speaking to the challenge associated with obtaining IgGs that neutralize viral infectivity is the fact that low representation of infectivity-neutralizing IgGs within repertoires obtained from human subjects has been observed for HIV and other viruses [21] outside the flavivirus family. One approach to enhancing the likelihood of identifying binders to more relevant epitopes on the virion surface might be to perform subsequent rounds of sorting in the presence of purified binding-but-not-neutralizing domains from earlier rounds of selection in order to “mask” viral epitopes that do not contribute to virus neutralization.

## Conclusions

Fluorescence-activated cells sorting of yeast surface-displayed Ab analogue library is an efficient method for de novo isolation of virion-binding domains. Affinities of isolated virion-binding domain clones are readily improved via FACS screening of mutant progeny libraries and high-affinity mutants identified in these screens can possess nanomolar virion binding affinities when expressed as soluble proteins. The procedures applied in this work can be extended to other yeast displayed binder libraries, particularly the immune libraries with paired heavy and light chains. Thus, FACS screening of yeast surface-displayed binding domain libraries against virions holds the potential to become a broadly utilized means of obtaining repertoires of virion-binding domains from which multispecific biopharmaceuticals for passive immunotherapy and prevention of viral infection can be constructed.

## Materials & methods

### Zika Virion production & purification

Five million Vero cells (Kidney epithelial cells from African green monkey, line CCL-81, ATCC) were plated per P150 culture dish and grown overnight at 37 °C in Dulbecco’s Minimum Essential Media (DMEM) supplemented with 10% Fetal Bovine Serum (FBS) and 1× Non-Essential Amino Acids (NEAA). The following day the cells were infected with Zika virions, strain PRVABC59 [30, 31] obtained from the CDC (GenBank Accession #KU501215), in 30 mL of fresh DMEM with 3% FBS and NEAA at a multiplicity of infection (MOI) of 0.1. After 5 days media was collected and cell debris was pelleted by centrifugation for 5 min at 500 rcf. Supernatant was passed through a 0.45 μm polyethersulfone (PES) filter (Fisher Scientific) and frozen at − 80 °C for subsequent ultrafiltration and buffer exchange or purification of Zika virions via ultracentrifugation over OptiPrep density gradient media.

Ultrafiltered, buffer-exchanged virions were used in yeast surface display binding experiments and ELISA assays. Thirty milliliters of virion-containing culture supernatant were thawed and split evenly between two VivaSpin Turbo 100 kDa MWCO ultrafiltration units (Sarstedt AG). Supernatants were concentrated to less than one mL per ultrafiltration (UF) unit via centrifugation at 2500 rcf for 1 hour. UF units were refilled to approximately 15 mL with pH 7.4 phosphate buffered saline (PBS), pH 7.4, containing 0.3% w/w BSA. UF units were centrifuged as above for an additional hour to a volume of less than 1 mL per unit, pooled, and the pool brought to a total volume of 2 mL by addition of PBS supplemented with 0.3% BSA.

To minimize safety concerns associated with handling of Zika virions used in yeast display and ELISA experiments virion activity was abolished via exposure to UV light in the presence of trimethylpsoralen hydrochloride (AMT) [32]. AMT (Sigma) was added to the above described two mL of concentrated virus-containing culture supernatant to a concentration of 10 μg/mL in a single well of a 24-well tissue culture plate. The plate, uncovered and within a biological safety cabinet, was placed six inches below an 8-watt UV lamp (Fisher Scientific) illuminating on the long wave setting for ninety minutes. Inactivated, ultrafiltered, buffer-exchanged virion preparations were stored at 4 °C and used in downstream experiments within 1 week of completion of the above preparatory procedures.

For ultracentrifugation-based purification of Zika virions to be used in flash sorting, three P150 culture dishes were seeded with Vero cells and infected at MOI 0.1 as described above. Infected Vero cell culture medium was harvested after 5 days and centrifuged as above. Pooled supernatant was concentrated approximately ten-fold to a final volume of less than 10 mL via 2000 rcf centrifugation for 40 min using the VivaSpin Turbo units noted above without any prior PES filtration step.

Immediately following concentration of cell culture supernatants UC purification of Zika virions [33] was carried out using allopolymer tubes (Denville Scientific) and OptiPrep density Gradient Media (Sigma). 15 mL of 12% OptiPrep in PBS (vol/vol) was overlaid on 10 mL of 35% OptiPrep (vol/vol) in 38.5 mL allopolymer tubes and 1.5 mL of concentrated viral supernatant was overlaid onto the 12% OptiPrep layer. Tubes were centrifuged overnight at 4 °C and 105,000 rcf in a Sorvall SureSpin 630 Ultraspeed rotor. After centrifugation, tubes were removed from the rotor and concentrated virus bands were visualized with an incandescent light bulb in a darkroom. A 21.5-gauge needle was used to puncture the allopolymer tubes and draw the concentrated Zika virion bands into a 3 mL syringe.

The approximately 3 mL of concentrated Zika virion preparation was loaded onto a VivaSpin Turbo unit along with 10 mL of PBS containing 0.05% BSA and concentrated at 2400 rcf for 20 min to a final volume of less than 1 mL. The VivaSpin unit was refilled to approximately 15 mL with PBS/0.05% BSA and centrifuged for an additional 25 min at 2400 rcf to a final volume of approximately 300 μL. Aliquots of this purified Zika virion preparation were frozen at − 80 °C for subsequent use in yeast-displayed Fn3 library sorting.

### Yeast surface-displayed Naïve Fn3 library FACS

One hundred and fifty milliliters of low-pH Sabouraud Dextrose Casamino Acid media (SDCAA, per liter - 20 g dextrose, 6.7 g yeast nitrogen base (VWR Scientific), 5 g Casamino Acids (VWR), Citrate buffer (pH 4.5) - 10.4 g sodium citrate / 7.4 g citric acid monohydrate) [11] was inoculated with strain EBY100 *Saccharomyces cerevisiae* displaying the naïve Fn3 library [13] to a starting optical density (OD) of 0.5 and shaken overnight at 250 rpm and 30 °C. The following day 5 mL Sabouraud Galactose Casamino Acid (SGCAA, Per liter - Phosphate buffer (pH 7.4) - 8.6 g NaH_2_PO*H_2_O / 5.4 g Na_2_HPO_4_, 20 g galactose, 6.7 g yeast nitrogen base, 5 g Casamino Acids) [11] induction cultures were started at an OD of 0.5 and shaken overnight at 250 rpm and 20 °C.

Prior to incubation with yeast, 200 μL of UC-purified Zika virion preparations were brought to a total volume of 700 μL in PBS/0.3% BSA and inactivated with 10 μg/mL AMT and long wave UV light as described above. After overnight induction, naïve Fn3 library yeast were harvested and two OD*mL (approximately twenty-million yeast cells, OD*mL equals culture optical density multiplied by milliliters of culture) were washed in PBS/0.3% BSA prior to resuspension in 600 μL of UC-purified Zika virion preparation diluted to a total volume of 1.5 mL in PBS/0.3% BSA; a two-mL capacity low-bind microcentrifuge tube (Fisher Scientific) was used for resuspension. Resuspended yeast were tumbled at 18 rpm overnight 4 °C using a tube rotator (VWR Scientific).

The following day yeast were pelleted for 30 s at 8000 rcf in a microcentrifuge, virion containing supernatant was aspirated off, and the yeast washed in PBS/0.3% BSA. For secondary labeling yeast were incubated in 1 mL of PBS/0.3% BSA containing 50 μg/mL of Alexa405-conjugated anti-*myc* IgY (Fisher) and 50 μg/mL Alexa488-conjugated anti-Zika virion human IgG (Adipogen). Alexa antibody conjugates were prepared using commercially available N-hydroxysuccinimide ester (NHS) coupling reagents (Fisher). During the 1 h secondary labeling incubation yeast were tumbled in a 2-mL low-bind tube as above in the dark at 4 °C.

All yeast FACS described in this work was performed on a MoFlo Astrios (Beckman Coulter) in the Texas A&M University Department of Veterinary Pathobiology. After secondary labeling yeast were washed in PBS/0.3% BSA as above and resuspended in one mL of PBS for FACS. Approximately 1.5 × 10^7^ yeast cell events were recorded in the first round of FACS (flash sort). For all FACS and flow cytometric analysis experiments yeast cells were resuspended in BSA-free PBS prior to being loaded onto the flow cytometer.

Yeast isolated during flash sorting were re-cultured in 3 mL SDCAA media with overnight shaking at 30 °C to an OD (600 nm) of 2.0 prior to overnight induction of a 5 mL SGCAA culture at 20 °C from a starting OD_600_ of 0.5. Prior to Round 2 of FACS, 60 μL of UC-purified virions were diluted in PBS/0.3% BSA and inactivated as above. The induced yeast that had been regrown after flash sorting were harvested, washed, and resuspended with UC-purified virions for overnight incubation as described above.

For Rounds 2 and 3 of naïve Fn3 library FACS a third fluorescent reagent, Alexa647-conjugated human IgG1 isotype control (BioLegend) was included in the secondary labeling buffer at 50 μg/mL. Inclusion of this fluorescent antibody facilitated simultaneous forward and counterscreening to enrich yeast displaying Fn3s that bind to Zika virions and exclude yeast displaying Fn3s that bind to human IgGs (Additional file 1: Figure S1).

Alexa405-positive/Alexa488 positive/Alexa647-negative yeast enriched in Round 2 of FACS were regrown in SDCAA and induced in SGCAA as described above. Virion preparation, yeast labeling and sorting procedures for Round 3 of FACS mirrored those of Round 2 with the exception that yeast isolated in the third round were serially diluted and plated on solid Synthetic Dropout minus tryptophan (SD -Trp) agar media [11] to facilitate both Fn3 gene sequencing and analysis of the virion binding properties of individual Fn3 clones via flow cytometry.

### Characterization of individual Fn3 clones enriched from Naïve library via FACS

The ZymoPrep Yeast Plasmid MiniPrep II Kit (Zymo Research) was used to obtain plasmid DNA from small amounts of colony material taken from each of twenty single yeast colonies that had grown up on the above noted SD -Trp agar plates. Plasmids were transformed into chemically competent NEB 5α *E. coli* (New England Biolabs), for clonal isolation and sequencing. Yeast-displayed Fn3 gene insert sequences were determined using respective upstream and downstream primers ConSqLt (5′ - CGTACTCTTTGTCAACGACTAC - 3′) and ConSqRt (5′ - CATGGGAAAACATGTTGTTTACG - 3′). These respective primers anneal upstream and downstream of the Fn3 gene inserts carried by the pCTCON2 backbone, respectively [11].

For assessment of individual yeast-displayed Fn3 clone binding to Zika virions, yeast colonies corresponding to unique Fn3 sequences were picked into 4 mL of SDCAA and grown overnight at 30 °C with shaking at 250 rpm. These cultures were subsequently induced in 5 mL of SGCAA overnight at 20 °C with shaking at 250 rpm; induction culture starting OD_600_ was 0.5. To achieve this starting OD_600_ yeast grown in SDCAA were pelleted by centrifugation followed by aspiration of SDCAA media supernatant and resuspension in SGCAA. Yeast were harvested and washed in PBS/0.3% BSA with pelleting performed by centrifugation for 30 s at 8000 rcf as described above.

All flow cytometric analysis experiments were performed using buffer-exchanged, ultrafiltered concentrated Zika virion preparations rather than UC-purified Zika virions; the latter virion preparations were used only for the three rounds of FACS carried out with the yeast-displayed naïve Fn3 library. Flow cytometric analyses were performed on a Fortessa analyzer (Becton Dickinson) in the Texas A&M College of Medicine Cell Sorting Facility.

The somewhat labor-intensive nature of generating Zika virion preparations for flow cytometry experiments motivated implementation of procedures intended to reduce the number of virions required to quantify binding interactions between virions and yeast-displayed Fn3s. For cases in which ligands are readily available, e.g., commercially purchased receptor proteins or growth factors that may be cancer therapy targets, concerns regarding amount of ligand used in yeast display flow cytometry studies are generally moderate or even nonexistent and the mixing of induced yeast with uninduced yeast, which is described below, is not practiced.

For flow cytometric analysis experiments 0.005 OD*mL (approximately 50,000 yeast cells), where 1 OD*mL equals the volume of yeast culture in 1 mL multiplied by optical density of the culture, of induced yeast were incubated with a ten-fold excess of uninduced yeast, which were grown overnight in SDCAA and harvested at the same time as induced yeast cultures, so that both ligand depletion and excessive loss of yeast cells during labeling and washing steps could be avoided. Yeast were incubated in either 150 μL or 750 μL of PBS/0.3% BSA with UF virion preparations being added at respective dilutions of 1:5 and 1:20; a larger PBS/0.3% BSA incubation volume was used in conjunction with the lower Zika virion concentration yeast incubations to further prevent ligand depletion effects.

Incubation of Fn3-displaying yeast with Zika virions, washing of yeast cells, and secondary labeling was carried out as described above for FACS with the exception that respective anti-*myc* and anti-Zika IgG labeling agents were coupled with different fluorophores to accommodate the different optical filter sets installed on the respective MoFlo and Fortessa instruments. For analysis studies carried out on the Fortessa Alexa488-conjugated anti-*myc* IgY was used to quantify Fn3 display and Alexa647-conjugated anti-Zika virion human IgG was used to measure Fn3 binding to Zika virions.

### Construction & screening of Zika Virion-binding site-directed mutant libraries

Site-directed Clone71 mutant libraries were constructed via overlap extension PCR and standard ligation using T4 DNA ligase (New England Biolabs). Oligonucleotide primers carrying NNB base triplets were purchased from Integrated DNA Technologies. The degenerate ‘B’ base is comprised of a mixture of C, G and T bases while the degenerate ‘N’ base is composed of all four nucleotide bases. Each pair of forward and reverse site-directed mutagenesis primers featured the NNB (forward) or VNN (reverse) base triplets at amino acid positions corresponding to Clone71’s antigen binding loop residues (Additional file 1: Figure S2). In the case of the Clone71 position 80/position 82 double site-directed mutant library the mutagenesis primer pair featured degenerate base triplets at two amino acid positions.

The ConSqLt and ConSqRt primers noted above were used as outer primers for Fn3 gene amplification reactions. Overlap extension PCR products were digested using NheI and BamHI (New England Biolabs) and ligated into the yeast surface display vector pCTCON2 [11] digested with these same two enzymes. Overnight ligation reactions were desalted using a DNA Clean & Concentrate-5 column (Zymo Research) and transformed into chemically competent NEB 5α cells.

For each transformation cells were plated onto three LB plates containing 100 μg/mL carbenicillin and after overnight incubation at 30 °C all colonies on plates for each respective transformation were scraped into 10 mL of LB + carbenicillin liquid media and grown overnight at 30 °C. Total colony counts for each single site-directed mutant library ranged from 500 to 2000; numbers considerably greater than the fewer than 50 colonies observed for a negative control ligation reaction that contained digested pCTCON2 backbone DNA absent any Fn3 gene insert. Ligation and transformation procedures were scaled up for the position 80/position 82 double site-directed mutant library so that more than 10,000 transformants were obtained.

DNA was harvested from overnight liquid cultures using the Qiagen Spin Miniprep Kit and transformed into the EBY100 yeast surface display strain [11] that had been made chemically competent using the Frozen EZ-Yeast Transformation II Kit (Zymo Research). For each single site-directed mutant library transformed yeast were plated onto three each SD -Trp plates and incubated at 30 °C for 2 days. 1000–3000 yeast transformants were obtained for each library. DNA preparation and yeast transformation for the position 80/position 82 double site-directed mutant library was scaled up to yield more than 10,000 EBY100 yeast transformants.

EBY100 yeast colonies were scraped off SD -Trp plates into 10 mL of low-pH SDCAA media and these master library cultures were diluted into culture tubes containing 5 mL of the same media to a starting OD_600_ of approximately 0.5. Three such tubes were prepared for the position 80/position 82 double site-directed mutant library. After overnight shaking at 30 °C and 250 rpm cultures were induced in SGCAA media as described above and subsequently harvested for initial screening using the Fortessa flow cytometer.

In these initial screens 0.05 OD*mL (approximately 500,000 yeast cells) of each induced yeast library were mixed with a ten-fold excess of uninduced yeast and resultant mixtures tumbled overnight at 4 °C in 200 μL of PBS/0.3% BSA to which 15 μL of UF Zika preparation had been added (approximately 15-fold virion prep dilution). Secondary labeling, washing, and resuspension in PBS were performed as described above. 250,000 yeast events were collected for each yeast-displayed Clone71 site-directed mutant library to assess the presence or absence of progeny mutants with Zika virion binding affinity potentially greater than that for the Clone71 parent.

The three site-directed mutant libraries containing clones with potentially increased Zika virion binding affinity were grown in SDCAA, induced, and harvested as described above. 0.2 OD*mL of each induced library were mixed with uninduced yeast at a 1:2 ratio to minimize ligand depletion and resultant yeast mixtures were tumbled overnight at 4 °C in PBS/0.3% BSA containing a ten-fold dilution of UF Zika virion preparation. Yeast populations enriched via FACS for each of the three Clone71 progeny mutant libraries were plated on SD -Trp plates and sequences for seven clones for each enriched population were determined as described above.

Relative Zika virion binding affinities for leading clones were determined via flow cytometric analysis. For these determinations the incubation conditions described above were adjusted both so that relative surface display levels for Fn3 clones could be compared absent any skewing caused by the presence of uninduced yeast and so that ligand depletion effects could be avoided. These objectives were achieved by incubating 0.015 OD*mL of each yeast clone with either 300 μL or 1.5 mL of PBS/0.3% BSA containing five-fold or twenty-fold dilutions of UF Zika virion preparation absent any uninduced yeast.

### Zika Virion-binding Fn3 soluble expression and purification

For soluble Fn3 expression in the cytoplasm of *E. coli* BL21 cells Fn3 genes were amplified by PCR using respective left and right primers NcoIFn3Lt (5′ -GATATACCATGGGCGAGCAGAAACTGATAAGTGAGGAAGATCTAGCTAGCTCCTCCGACTCTCCGCGTAACCTGGAGGTTAC - 3′) and HndIIIFn3Rt (5′ - CAGTTCGGATCCTCATTAATGGTGATGGTGATGGTGCTGAGACGGTTTGTCGATTTCGGTGCGATAATTG - 3′) and the PCR products were digested with NcoI and HindIII (New England Biolabs). The NcoIFn3Lt primer appends the solubly expressed Fn3s with N-terminal *myc* tags whereas the HndIIIFn3Rt primer places a His_6_-tag at the Fn3 C-terminus. Digested PCR products were ligated into NcoI-HindIII digested pET28a vector (Novagen) in standard ligation reactions with T4 DNA ligase. Ligation reactions were desalted and transformed into chemically competent NEB 5α *E. coli* cells and plated on LB agar plates containing 50 μg/mL kanamycin. Plasmid DNA was harvested as described above and plasmids carrying correct Fn3 gene inserts transformed into competent BL21(DE3) *E. coli* cells (New England Biolabs) with plating onto LB agar with 50 μg/mL kanamycin; plates were incubated overnight at 33 °C to prevent colony overgrowth.

Soluble expression of Fn3s was carried out under autoinducing conditions. A single colony from a freshly transformed or streaked LB agar plate was used to inoculate 5 mL of autoinduction media (per Liter - Phosphate buffer (pH 7.2): 6 g Na_2_HPO_4_ / 3 g KH_2_PO_4_, 20 g tryptone, 5 g yeast extract, 5 g NaCl, 50 mg kanamycin) and inoculated tubes were shaken at 240 rpm for 20–24 h at 37 °C. Three tube cultures were grown up for each Fn3.

Each 5 mL culture was pelleted individually by centrifugation for 5 min at 2500 rcf and subsequently frozen at − 80 °C or immediately processed to extract the expressed Fn3s. Cell pellets were lysed by incubation in pH 7.4 PBS containing 200 μg/mL hen egg lysozyme (Sigma) and a 1:400 dilution of Lysonase bioprocessing reagent (EMD Millipore) for 45 min at 37 °C followed by two freeze-thaw cycles using liquid nitrogen as the cryogenic agent. After bringing to final volume of 1 mL by addition of PBS and centrifugation at 18,000 rcf for 10 min supernatants were tumbled head-over-head in microfuge tubes with 30 μL of Qiagen Ni-NTA resin for 1 h at room temperature. Resin was subsequently washed with 1 mL PBS and 1 mL PBS/15 mM imidazole prior to Fn3 elution via 15-min incubation of resin in 200 μL of PBS/150 mM imidazole. Eluted Fn3s were buffer exchanged into PBS using Zeba Spin desalting columns (Fisher Scientific) and Fn3 protein concentrations determined using the Pierce Coomassie Protein Assay kit (Fisher Scientific).

PAGE analysis of purified Fn3s was carried out under both denaturing and nondenaturing conditions using a BioRad Mini-Protean Tetra Cell electrophoresis system. Denaturing PAGE analysis was performed using 4% stacking/12% resolving Tris-HCl gels. Between 0.5 and 1 μg of purified, desalted Fn3 was loaded into each well and electrophoresis was performed at 200 V for 35 min. EZ Run Rec Unstained Protein Ladder (Fisher Scientific) was used as a molecular weight standard. Gels were stained with Coomassie Blue (Fisher Scientific) following electrophoresis.

Native PAGE analysis was carried out using 3% stacking/12% resolving Bis-Tris gels with the 0.5–1.0 μg of purified Fn3 noted above loaded into each well. Two electrophoresis intervals were carried out, an initial 15-min interval at 100 V followed by a 55-min interval at 200 V. Gels were stained with Coomassie Blue following electrophoresis.

### ELISA detection of soluble Fn3s binding to Zika Virions

For assays measuring binding of immobilized Fn3s to Zika virions, purified, desalted Fn3s were diluted to 8 μg/mL in pH 7.4 PBS and adsorbed onto MaxiSorp 96-well plates (BioLegend) by overnight incubation of 100 μL of Fn3-containing solution at 4 °C. The following morning Fn3-containing solutions were aspirated off and microplate wells were filled with 300 μL of blocking buffer (PBS containing 2% BSA) and incubated with rocking for 1 hour at room temperature. Blocked wells were subsequently washed four times with 200 μL of PBS prior to incubation with UF Zika virion preparations.

UF Zika virion preparations were diluted 1:3 in blocking buffer. Conditioned culture medium from uninfected Vero cells, concentrated using VivaSpin Turbo units as described above for virion-containing culture supernatants, was used as a negative control and was diluted 1:3 in blocking buffer. One-hundred μL of either UF virion preparation or concentrated conditioned medium were applied to each well and incubated with rocking for ninety minutes at room temperature. Wells were washed with PBS as above then 100 μL of the above-described anti-Zika human IgG diluted to 2 μg/mL in blocking buffer were applied to each well followed by rocking for 45 min at room temperature. After four additional washes 100 μL of horseradish peroxidase (HRP)-conjugated goat anti-human IgG (Jackson ImmunoResearch) diluted 1:10000 in blocking buffer were applied to each well with subsequent rocking for thirty-five minutes at room temperature.

After incubation with HRP-conjugated anti-human IgG wells were washed five times with 300 μL of PBS and 100 μL of Pierce UltraTMB substrate (Fisher Scientific) were added to each well. Substrate development was halted after thirty minutes via addition of 100 μL of 1 M H_2_SO_4_ and binding of immobilized Fn3s to Zika virions quantified by measuring absorbance at 450 nm on a Tecan Infinite M200 plate reader.

For assays measuring binding of free-in-solution Fn3s to immobilized Zika virions anti-Zika human IgG was diluted to 200 μg/mL in PBS and adsorbed onto MaxiSorp 96-well plates by overnight incubation of 100 μL of IgG-containing solution at 4 °C. The following morning IgG-containing solutions were aspirated off and microplate wells were filled with 300 μL of blocking buffer and incubated with rocking for 1 hour at room temperature; negative control wells that had been incubated overnight with PBS sans anti-Zika IgG were included in this blocking step. Blocked wells were subsequently washed four times with 300 μL PBS prior to overnight incubation at 4 °C with 100 μL of UF Zika virion preparations or negative control concentrated Vero cell culture supernatant diluted 1:3 in blocking buffer.

After overnight incubation of IgG-coated plates with Zika virions wells were washed four times with 300 μL of PBS. One hundred μL of purified, desalted Fn3 that had been diluted to the desired concentration in blocking buffer were added to each well with subsequent incubation at room temperature for ninety minutes. Following four washes with 300 μL of PBS, 100 μL of 200 ng/mL solution of anti-*myc* IgY in blocking buffer were added to each well with subsequent rocking for 1 hour at 4 °C.

After incubation with anti-*myc* IgY wells were washed as above and 100 μL of anti-IgY HRP diluted to 100 ng/μL in blocking buffer was added to each well. Following rocking for forty minutes at 4 °C wells were washed five times with 300 μL of PBS and 100 μL of Pierce UltraTMB substrate were added to each well. Substrate development was halted after fifteen minutes via addition of 100 μL of 1 M H_2_SO_4_ and binding of Fn3s to Zika virions quantified by measuring absorbance at 450 nm on a Tecan Infinite M200 plate reader.

### Melting temperature determination of Fn3 variants

Freshly IMAC-purified Fn3 proteins F80P, I82Y and F80P/I82Y were diluted to 5 μM in PBS before being mixed with SYPRO orange solution (final concentration 20×) in PCR tubes at room temperature. These samples were immediately transferred to a CFX96 qPCR instrument and heated from 25 to 95 °C (0.5 °C per step, hold 30s at each step). The fluorescence signal in the FRET channel was recorded in order to obtain the melting curve for each sample. The melting temperature (Tm) was determined based on the midpoint of the melting curve using CFX Manager software (BioRad).

## Supplementary information

Additional file 1: Contains Table S1 and Figures S1-S3. (DOCX 566 kb)

## Data Availability

The datasets used and/or analyzed during the current study are available from the corresponding author on reasonable request.

## Abbreviations

Ab: antibody
AMT: trimethylpsoralen hydrochloride
BSA: bovine serum albumin
DARPin: designed ankyrin repeat protein
DMEM: Dulbecco’s Minimal Essential Medium
EC_50_: half maximal effective concentration
Fab: antibody antigen-binding fragment
FBS: fetal bovine derum
Fn3: Type III human fibronectin domain
Fv: antibody fragment variable region
HRP: horseradish peroxidase
IgG: Immunoglobulin G
MOI: multiplicity of Infection
NEAA: nonessential amino acids
OD: optical density
PBS: Phosphate Buffered Saline
PES: polyethersulfone
scFv: Single chain variable fragment
SD -Trp: Synthetic Dropout minus Tryptophan
SDCAA: Sabouraud Dextrose Casamino Acid
SGCAA: Sabouraud Galactose Casamino Acid
Tm: melting temperature
TMB: 3,3′,5,5′-Tetramethylbenzidine
UC: Ultracentrifugation
UF: Ultrafiltration
V_H_H: Single domain camelid antibody
