# Supplementary material for: De Novo Isolation & Affinity Maturation of yeast-displayed Virion-binding human fibronectin domains by flow cytometric screening against Virions
Source: J Biol Eng. 2019 Oct 16;13:76. doi: 10.1186/s13036-019-0203-2 (PMC6796422; doi:10.1186/s13036-019-0203-2)
Supplement: Supplementary file 1 — Contains Table S1 and Figures S1-S3. (DOCX 566 kb) [file 13036_2019_203_MOESM1_ESM.docx]

**Additional File 1 - Text S1**

Sequences for Zika virion-binding, negative control, and wild type Fn3s. Binding loop residues underlined in bold. Positions 80 and 82 for Clone 71 mutant highlighted in yellow.

Clone51

A S S S D S P R N L E V T N A T P N S L T I S W **D D H A S A V Y** Y R I T Y G E T G G N S P S Q E F T V P **G T S Y S** A T I S G L K P G Q D Y T I T V Y **A V T G G N P F S N** P I S I N Y R T

Clone71

A S S S D S P R N L E V T N A T P N S L T I S W **D A P Y E T A Y G** Y R I T Y G E T G G N S P S Q E F T V P **G T N N N** A T I S G L K P G Q D Y T I T V Y **A V S S F N I W G S N** P I S I N Y R T

Negative Control

A S V S D V P R D L E V V A A T P T S L L I S W **F D Y A V T Y** Y R I T Y G E T G G N S P V Q E F T V P **G W I S T** A T I S G L K P G V D Y T I T V Y A V T **D N S R W P F R S** T P I S T N Y R T

Wild Type Fn3

A S V S D V P R D L E V V A A T P T S L L I S W **D A P A V T V R Y** Y R I T Y G E T G G N S P V Q E F T V P **G S R S T** A T I S G L R P G V D Y T I T V Y A V T **G R D G S P A S S R** P I S I N Y R T

Residues underlined in non-boldface type in the Negative Control and Wild Type Fn3 sequences were mutated to more hydrophilic amino acids for construction of the naïve Fn3 library from which Clone51 and Clone71 were isolated [1]. These substitutions were introduced to make the Fn3 scaffold less susceptible to engaging in nonspecific interactions with off-target proteins [2]. The Negative Control Fn3 was obtained from a yeast surface-displayed Fn3 library [3] comprised of clones that do not contain these hydrophilic scaffold residue substitutions.

**Additional File 1 - Figure S1**

**Figure S1. (A)** Schematic for simultaneous forward/counterscreening to prevent enrichment of Fn3s binding to detection reagents during FACS. **(B)** Flow Cytometry Dot Plots. Left: Y-axis denotes binding to Alexa647-labeled human IgG isotype control, X-axis denotes Fn3 surface display. Dark red sort gate indicates Alexa647-negative yeast that are analyzed in right panel dot plot. Right:: Y-axis denotes binding to Zika virions as quantified via sandwich detection using Alexa488-conjugated anti-Zika mAb, X-axis denotes Fn3 surface display. Yeast cells falling within purple rectangle in right plot are human IgG isotype control-negative/Zika virion-positive and are isolated for regrowth and additional FACS. Flow cytometer generates both dot plots simultaneously.

**Additional File 1 - Figure S2**


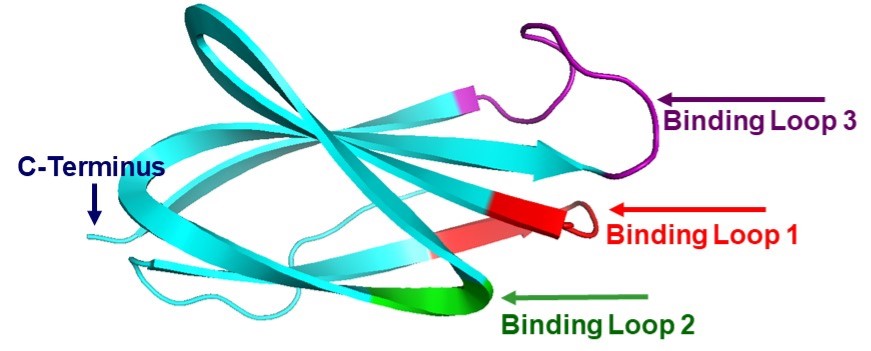


**Figure S2.** Ribbon representation of Fn3 structure (PDB 2DM4).

**Additional File 1 - Figure S3**

**Figure S3. Representative flow cytometry dot plots for FACS isolation of high-affinity clones from Clone71 mutant libraries.** Y-axes denote binding of surface-displayed Fn3s to Zika virions as quantified via sandwich detection using Alexa488-conjugated anti-Zika IgG. X-axes denote Fn3 surface display. Yeast lying within the orange polygons were regrown and plated with individual colonies being picked to assess Zika virion binding affinity. Percentages of displaying yeast populations isolated via FACS denoted in orange. Induced yeast were mixed with uninduced yeast at a ratio of 1:2 to prevent ligand (Zika virion) depletion effects. Dot plots contain data for 100,000 analyzed yeast cells.

**References:**

1) Woldring DR, Holec PV, Zhou H, Hackel BJ. High-Throughput Ligand Discovery Reveals a Sitewise Gradient of Diversity in Broadly Evolved Hydrophilic Fibronectin Domains. PLoS One. 2015;10(9):e0138956.

2) Hackel BJ, Sathirachinda A, Gambhir SS. Designed hydrophilic and charge mutations of the fibronectin domain: towards tailored protein biodistribution. Protein Eng Des Sel. 2012; 25(10):639-47.

3) Hackel BJ, Kapila A, Wittrup KD. Picomolar affinity fibronectin domains engineered utilizing loop length diversity, recursive mutagenesis, and loop shuffling. J Mol Biol. 2008;381(5):1238-52.
